# Sarcoïdose à localisation hépatosplénique : description d'un cas au Centre hospitalier universitaire de Brazzaville, Congo

**DOI:** 10.48327/mtsi.v4i1.2024.478

**Published:** 2024-02-05

**Authors:** Alexis ELIRA DOKEKIAS, M. R. ADEGBINNI AKANDE, Firmine Olivia GALIBA ATIPOTSIBA, Lydie OCINI NGOLET, Richard MIKOUIYI NGOULOU, Jennifer ELIRA SAMBA, Didace MASSAMBA MIABAOU, Donatien MOUKASSA

**Affiliations:** 1 Service d'hématologie clinique, CHU Brazzaville, et Centre national de référence de la drépanocytose « Antoinette SASSOU NGUESSO », Brazzaville, République du Congo; 2 Service de chirurgie digestive, CHU Brazzaville, République du Congo; 3 Laboratoire d'anatomie et cytopathologie, Polyclinique internationale de Brazzaville, République du Congo

**Keywords:** Granulomatose, Sarcoïdose, Rate, Foie, Brazzaville, République du Congo, Afrique subsaharienne, Granulomatosis, Sarcoidosis, Spleen, Liver, Brazzaville, Congo Republic, SubSaharan Africa

## Abstract

La sarcoïdose est une maladie inflammatoire multisystémique d’étiologie inconnue. La forme extrapulmonaire isolée est rare. Nous rapportons le cas d'une sarcoïdose hépatosplénique chez une patiente de 29 ans.

Il s'agit d'une patiente sans antécédents médicaux notables, qui a été reçue en consultation pour des épistaxis répétées. L'examen clinique a noté une hépatomégalie nodulaire associée à des signes d'hypertension portale et une splénomégalie. La vitesse de sédimentation, les phosphatases alcalines, le taux sérique de l'enzyme de conversion de l'angiotensine, les aminotransférases étaient élevées. L'examen histologique de la rate et de la biopsie hépatique a noté une infiltration inflammatoire granulomateuse sans lésion cancéreuse et sans caséum. Ce tableau est compatible avec une sarcoïdose malgré l'absence de PET-Scan. Le principal défi reste le diagnostic différentiel avec les autres granulomatoses. La corticothérapie est le traitement de première intention ayant permis après splénectomie une stabilité clinique et biologique.

## Abstract (en)

Sarcoidosis is a multisystem inflammatory disease of unknown etiology. The isolated extrapulmonary form is rare. We report the case of hepatosplenic sarcoidosis in a 29-year-old female patient.

It is a patient with no notable medical history, who was seen in consultation for repeated epistaxis. Clinical examination noted nodular hepatomegaly associated with signs of portal hypertension and splenomegaly. Sedimentation rate, alkaline phosphatase, serum angiotensin converting enzyme, aminotransferases were high. Histological examination of the spleen and liver biopsy noted granulomatous inflammatory infiltration without cancerous lesion or tonsil stones.

This picture is comparable with sarcoidosis, despite the absence of PET scans. The main challenge remains the differential diagnosis with other granulomatoses. Corticosteroid therapy is the first-line treatment, and after splenectomy the patient has achieved clinical and biological stability.

## Introduction

La sarcoïdose ou maladie de Besnier-BoeckSchaumann est une maladie multisystémique, d’étiologie inconnue, caractérisée par la formation de granulomes épithélioïdes et gigantocellulaires dépourvus de nécrose caséeuse [4, 5, 8]. Tous les organes peuvent être atteints, cependant l'atteinte pulmonaire est la présentation la plus courante. Le foie est l'un des organes les plus fréquemment touchés après les poumons et les ganglions lymphatiques [7]. La sarcoïdose est répandue dans le monde entier, avec une prévalence plus élevée dans les pays nordiques et chez les Afro-américains aux États-Unis [4, 5]. Les patients âgés de 20 à 40 ans sont les plus atteints [5]. Nous rapportons le cas d'une sarcoïdose à localisation hépatosplénique chez une jeune femme.

## Observation clinique

Patiente de 29 ans, congolaise, sans antécédents notables, reçue en consultation en juin 2022 pour une épistaxis bilatérale de moyenne abondance survenue de façon spontanée. Elle présente depuis plus de 3 ans un ballonnement abdominal progressif avec une sensation de pesanteur intra-abdominale, un amaigrissement et une hypersudation. Le diagnostic d'une cirrhose avait été initialement retenu. L'examen clinique a noté une patiente consciente, légèrement ictérique et un bon état hémodynamique. Le foie était augmenté de taille avec une flèche hépatique à 17 cm, indolore, ferme, à surface irrégulière. Il y avait une splénomégalie mesurant 16 cm. La rhinoscopie antérieure, l'auscultation cardiaque et pulmonaire étaient normales.

Les explorations paracliniques retrouvaient une cytolyse avec les ASAT à 421,5 Ul/l, les ALAT à 204,3 Ul/l, et une cholestase avec les phosphatases alcalines à 670 Ul/l. Le taux de bilirubine était augmenté (50,4 µmol/l) aux dépens de la forme conjuguée (30,8 µmol/l). La vitesse de sédimentation était à 150 mm à la première heure, et le taux sérique de l'enzyme de conversion de l'angiotensine (ECA) était augmenté à 130 Ul/l. L'hémogramme montrait une anémie modérée microcytaire et hypochrome. L'intradermoréaction (IDR) à la tuberculine était négative et le taux d'alphafœtoprotéine était normal. La créatininémie, le bilan d'hémostase, la ferritinémie et l’électrophorèse des protéines sériques étaient normaux. Les sérologies virales étaient négatives (Ag HbS, Anticorps anti-VHC) indiquant une absence d'hépatite B et C.

**Figure 1:**
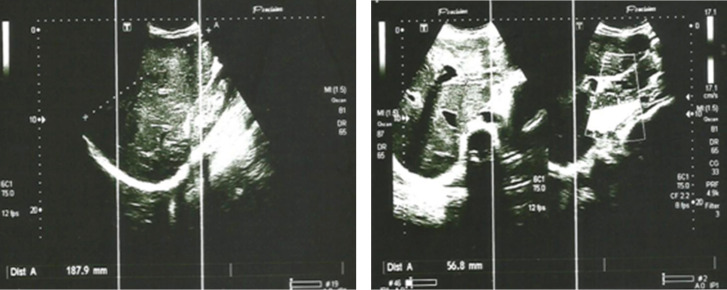
Échographie abdominale : hépatomégalie à contours micro-bosselés avec hypertrophie du segment I (A et B) en rapport avec des stigmates d'hépatopathie chronique débutante Abdominal ultrasound: hepatomegaly with micro bumpy contours with hypertrophy of segment I (A and B) related to signs of early chronic liver disease

L’échographie abdominale a mis en évidence une hépatomégalie nodulaire, une splénomégalie, une ascite de faible abondance et une dilatation de la veine porte (Fig. 1A et B).

La radiographie thoracique de face était normale.

Lexploration peropératoire pour splénectomie a mis en évidence une volumineuse splénomégalie de 18 cm bosselée, une hépatomégalie multi-nodulaire, des vaisseaux spléniques et portes très dilatés. Il n'y avait pas d'adénopathies.

**Figure 2:**
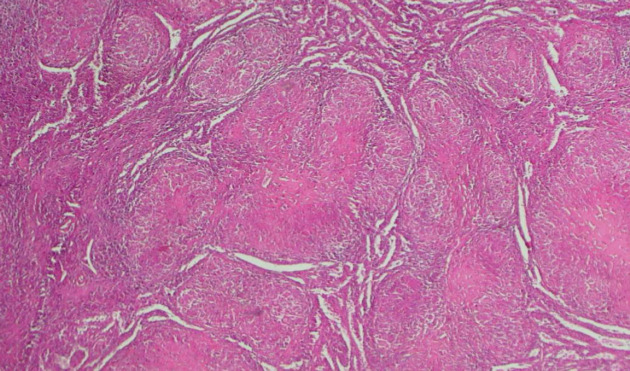
Granulome inflammatoire non nécrosant, centré par du collagène hyalinisé (HE × 4) Non-necrotizing inflammatory granuloma, centered by hyalinized collagen (HE × 4)

L'examen histologique de la biopsie hépatique a noté des signes d'inflammation chronique sans signe de cirrhose. L'examen histologique de la pièce de splénectomie a noté des remaniements hémorragiques. La microscopie a révélé un granulome inflammatoire sans nécrose caséeuse (Fig. 2 et 3), en faveur d'une sarcoïdose hépatique et splénique. Cependant, nous n'avons pas pu réaliser d'analyses immunohistochimiques complémentaires.

**Figure 3:**
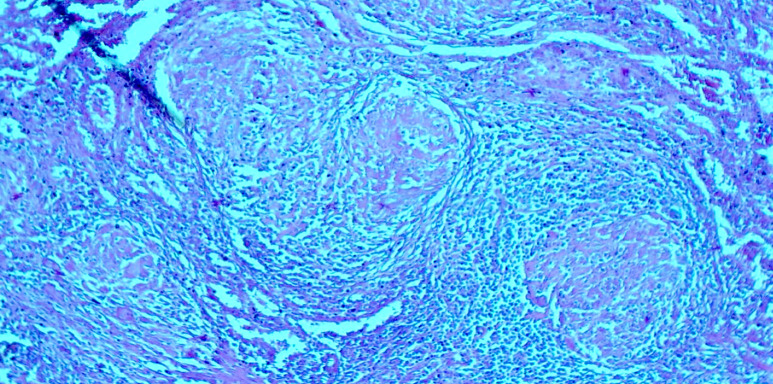
Couronne lymphocytaire des granulomes non nécrosants (HE × 10) Lymphocyte crown of non-necrotizing granulomas (HE × 10)

Le traitement a consisté en une corticothérapie par voie orale à raison de 60 mg de prednisone par jour puis réduite à moins de 20 mg par jour et une supplémentation martiale (200 mg par jour).

L’évolution clinique et paraclinique à plus de 18 mois est satisfaisante.

## Discussion

La sarcoïdose à localisation hépatique et splénique est une affection rare [8]. Au Congo, les atteintes hépatospléniques rapportées étaient associées à une atteinte pulmonaire [3]. À notre connaissance, il s'agit du premier cas de sarcoïdose hépatosplénique isolée rapporté au Congo. Les vomissements, la douleur de l'hypochondre droit et la cholestase anictérique constituent le mode de révélation le plus fréquent [3]. La ponction biopsie hépatique (PBH), qui permet le diagnostic positif, met en évidence un granulome épithélio-gigantocellulaire sans nécrose caséeuse. Le caractère invasif de la PBH limite sa réalisation [1]. Ainsi le diagnostic de la sarcoïdose reste le plus souvent présomptif, évoqué devant un faisceau d'arguments cliniques et paracliniques. Le PET-Scan paraît être un outil intéressant et non invasif pour le diagnostic de la sarcoïdose viscérale [1].

Le principal défi du clinicien est de poser le diagnostic différentiel avec les autres granulomatoses à localisations hépatique et/ou splénique. Concernant leurs étiologies, elles sont variées et de répartition différente selon la zone géographique. Les causes auto-immunes prédominent dans les pays développés et l'origine infectieuse, notamment la tuberculose hépatosplénique, dans les pays en voie de développement [6]. Dans notre cas, l’évolution chronique, l'absence de fièvre et la négativité de l'IDR à la tuberculine ne plaidaient pas en faveur de la tuberculose. L'histoplasmose hépatique donne un tableau clinique similaire à celui d'une sarcoïdose [2]. En outre, l'absence de nécrose caséeuse et de calcification était plus évocatrice d'une sarcoïdose.

La corticothérapie est le traitement de première intention dans les formes symptomatiques [3]. Elle permet le plus souvent une rémission clinique, biologique et radiologique [5]. La disponibilité et l'accessibilité de cette thérapeutique facilitent la prise en charge en Afrique. Des résultats satisfaisants ont été observés après plus de 18 mois de suivi. D'autres moyens thérapeutiques, non utilisés dans notre cas, ont montré leur efficacité. Il s'agit d'antipaludéens de synthèse en association avec l'acide ursodésoxycholique ou les autres immunosuppresseurs. La transplantation hépatique est réservée aux cas avancés avec une altération significative de la fonction hépatique [5, 9].

## Conclusion

Le diagnostic de la sarcoïdose hépatosplénique repose sur un faisceau d'arguments cliniques, biologiques et radiologiques. La ponction biopsie hépatique et splénique peut, en dehors de contre-indications, permettre le diagnostic histologique. La sarcoïdose est probablement sous-diagnostiquée en Afrique et sa prise en charge en général tardive. La corticothérapie et l'acide ursodésoxycholique constituent le traitement de première ligne chez les patients symptomatiques.

## Contribution des auteurs

Alexis ELIRA DOKEKIAS : auteur principal Rachad ADEGBINNI AKANDE, Firmine Olivia GALIBA ATIPO-TSIBA, Lydie OCINI NGOLET, Richard MIKOUIYINGOULOU, Jennifer ELIRA SAMBA : spécialistes ayant pris en charge la patiente

Didace MASSAMBA MIABAOU : chirurgien ayant pratiqué l'intervention

Donatien MOUKASSA : anatomopathologiste ayant fait le diagnostic

## Conflits d'intérêts

Les auteurs ne déclarent aucun conflit d'intérêts.
